# Early detection of cardiac involvement in Miyoshi myopathy: 2D strain echocardiography and late gadolinium enhancement cardiovascular magnetic resonance

**DOI:** 10.1186/1532-429X-12-31

**Published:** 2010-05-24

**Authors:** E Ryoung Choi, Sung-Ji Park, Yeon Hyeon Choe, Dong Ryeol Ryu, Sung-A Chang, Jin-Oh Choi, Sang-Chol Lee, Seung Woo Park, Byoung Joon Kim, Duk-Kyung Kim, Jae K Oh

**Affiliations:** 1 Cardiovascular Imaging Center, Cardiac and vascular center, Samsung Medical Center, Sungkyunkwan University School of Medicine, 50 Irwon-dong, Gangnam-gu, Seoul, 135-710, Seoul, Republic of Korea; 2 Department of Neurology, Sungkyunkwan University School of Medicine, 50 Irwon-dong, Gangnam-gu, Seoul, 135-710, Seoul, Republic of Korea

## Abstract

**Background:**

Miyoshi myopathy (MM) is an autosomal recessive distal myopathy characterized by early adult onset. Cardiomyopathy is a major clinical manifestation in other muscular dystrophies and an important prognostic factor. Although dysferlin is highly expressed in cardiac muscle, the effect of dysferlin deficiency in cardiac muscle has not been studied. We hypothesized that early myocardial dysfunction could be detected by 2D strain echocardiography and late gadolinium enhancement (LGE) cardiovascular magnetic resonance (CMR).

**Method:**

Five consecutive MM patients (3 male) in whom we detected the DYSF gene mutation and age-matched healthy control subjects were included. None of the patients had history of cardiac disease or signs and symptoms of overt heart failure. Patients were studied using 2D strain echocardiography and CMR, with 2D strain being obtained using the Automated Function Imaging technique.

**Results:**

All patients had preserved left ventricular systolic function. However, segmental Peak Systolic Longitudinal Strain (PSLS) was decreased in 3 patients. Global PSLS was significantly lower in patients with MM than in control subjects (p = 0.005). Basal anterior septum, basal inferior septum, mid anterior, and mid inferior septum PSLS were significantly lower in patients with MM than in control subjects (P < 0.0001, < 0.0001, 0.038 and 0.003, respectively). Four patients showed fibrosis by LGE. The reduced PSLS lesion detected by 2D strain tended to be in the same area as that which showed fibrosis by LGE.

**Conclusions:**

Patients with MM showed subclinical involvement of the heart. 2D strain and LGE are sensitive methods for detecting myocardial dysfunction prior to the development of cardiovascular symptoms. The prognostic significance of these findings warrants further longitudinal follow-up.

## Background

Miyoshi Myopathy (MM) is a distinct form of muscular dystrophy caused by mutations within the dysferlin (*DYSF*) gene resulting in severe to complete deficiency of dysferlin expression[1,2]. Clinically, these dysferlinopathies start in young adulthood with progressive muscle weakness and atrophy that advances to severe disability in older adulthood. While the profound effect of dysferlin deficiency in skeletal muscle has been the subject of much investigation, the effect of dysferlin deficiency in cardiac muscle have not been studied yet. Kuru et al[3] reported a 57-year-old Japanese woman with dysferlinopathy manifested as secondary dilated cardiomyopathy attributable to muscular dystrophy. Recently, Wenzel et al[4] described that 2 of 7 patients with dysferlinopathy who had progressive shortness of breath had dilated cardiomyopathy. Five other dysferlinopathy patients who had no cardiac symptoms had other cardiac abnormalities such as left ventricular hypertrophy and repolarization abnormalities. These observations suggest that dysferlin deficiency can lead to cardiomyopathy as well as to muscular dystrophy. Therefore, this study aimed to detect early cardiac involvement in patients with asymptomatic MM by using 2D strain by echocardiography (echo) and late gadolinium enhancement (LGE) by cardiovascular magnetic resonance (CMR).

## Methods

### Study population

Five consecutive index patients (three male and two female) in whom a DYSF gene mutation and distal myopathy was documented gave written informed consent. Five healthy subjects (mean age 43.8 ± 12.7 years) were enrolled as age-matched controls. The institutional review board of the Samsung Medical Center approved this study. The recommendations of the revised version of the Declaration of Helsinki were met. Cardiac involvement was investigated with conventional methods using standard 12-channel surface-electrocardiography (ECG), transthoracic echocardiography, and CMR.

### Transthoracic echocardiography

Transthoracic echocardiograms were performed with a commercially available echocardiographic instrument (Vivid 7, GE Medical Systems, Milwaukee, WI). A standard comprehensive M-mode, 2-D echocardiogram and echocardiographic Doppler studies were performed. Examinations included measurements of left ventricular (LV) volume and ejection fraction (EF), using the biplane disc method, and the left atrial volume index. All measurements were made according to the recommendations of the American Society of Echocardiography. Mitral inflow was obtained by pulsed-wave Doppler echocardiography, with the sample volume between mitral leaflet tips during diastole, and mitral annulus velocities were obtained from the septal and lateral annulus by tissue Doppler imaging. All measurements were performed over three cardiac cycles and then averaged.

### Strain measurement using the automated function imaging technique (AFI)

Global LV longitudinal strain was assessed using the automated function imaging (AFI) technique, which provides a new imaging modality based on 2-dimensional longitudinal strain imaging[5-7]. Longitudinal strain (percentage) is defined as the physiologic change in length of the region of interest from end-diastole to end-systole. During this period, strain in the longitudinal direction is a negative value as the length of the region of interest decreases. Longitudinal strain can be calculated using the following formula: longitudinal strain (%) = [L (end-systole) - L (end-diastole)]/L (end-diastole) × 100%; where L is the length of the region of interest. Assessment of global LV longitudinal strain (AFI global LV strain; described by the software as GLPS_Avg) provides 1 value representing the overall peak systolic longitudinal strain of all individual LV segments. With the commercially available AFI technique (General Electrics, Milwaukee, Wisconsin, USA), myocardial tissue deformation (strain) is calculated using speckle tracking from 2-dimensional grey scale images. For this analysis, a set of three longitudinal 2-dimensional image planes (apical long-axis, 2- and 4-chamber views) was used. Aortic valve closure timing was marked (to determine the end of systole) in the selected views and three points were anchored inside the myocardial tissue, two placed at the basal segments along the mitral valve annulus and one at the apex. These points triggered the automatic process, which analyzed myocardial motion by tracking features (natural acoustic tags). The percent of wall lengthening and shortening was displayed for each plane, representing longitudinal strain. The results of all three planes were then combined in a single bull's-eye summary, which presents the analysis for each segment along with a global strain value for the LV. For global LV longitudinal strain analysis, digital cine-loops were off-line processed using commercially available software (EchoPac 6.1, GE Medical Systems, Horten, Norway). All images were obtained at a frame rate of 50-80 frames/sec without dual focus. Three consecutive cardiac cycles were saved in digital format for off-line analysis. Investigators who were blinded to clinical features or CMR findings performed the strain analysis.

### Cardiovascular magnetic resonance (CMR)

All patients were scanned using a 1.5-T scanner (Achieva 1.5 T; Philips Medical Systems, Best, The Netherlands). Criteria for exclusion from the study included generally accepted contraindication to CMR, but no patient had to be excluded. CMR focused on the detection of fibrosis (LGE). Volumetric evaluation of LV mass, volume, and EF by manual tracing were performed. Various pulse sequences were used for imaging. ECG-gated double inversion-recovery images (TE = 10 ms), triple inversion-recovery images (TE = 100 msec), transverse or short-axial breath-hold cine MR images (balanced turbo field echo [TFE], TR/TE = 3.1/1.6 ms) were usually obtained before contrast injection. After injection of contrast material (gadobutrol at 0.1-0.15 mmol/kg at a rate of 3 mL/s, Gadovist; Bayer Schering Pharma, Berlin, Germany), post injection images were obtained using late myocardial imaging (T1 fast field echo, TR/TE = 4.5/1.4 ms) with suppression of the normal myocardial signals 5, 10, and 15 minutes later. A Look-Locker sequence was applied to determine optimal inversion time for myocardial nulling before delayed myocardial imaging in every instance.

### Statistical analysis

Data are reported as mean ± standard deviation. We used unpaired 2-sided t test to assess differences between groups (patients with MM vs controls) for normally distributed continous data. Parametric Man-Whitey test were used if data were nor normally distributed. All analyses were performed using commercially available software (SPSS version 10.0, SPSS Inc., Chicago, IL, and JMP, SAS Institute Inc., Cary, NC) and a p value < 0.05 was considered significant.

## Results

### Patient characteristics

The history, laboratory, and clinical data of the patients are shown in Table 1. None of the patients had a cardiac history or signs and symptoms of overt heart failure such as dyspnea on exertion or peripheral edema. All the patients experienced early adulthood onset of slowly progressive muscle weakness that preferentially involved muscles in the lower extremities. Creatine kinase (CK) levels were characteristically elevated in all patients.

**Table 1:** History, clinical and laboratory finding of patients

		#1	#2	#3	#4	#5
Age		30	42	35	62	50
sex		M	M	F	M	F
Other risk factor		None	DM/HTN	None	DM/HTN	none
Blood pressure (mmHg)		130/74	169/115	128/82	91/64	116/73
Heart rate (bpm)		69	99	73	70	65
Lab	CK(IU/l)	4822	1794	3520	528	2362
	NT-pro BNP(pg/mL)	29.27	20.93	34.75	156.2	18.62
DYSF mutation		c.[1284+2T>C]+[2997G>T]	c.3307A>T,c.4201insC	c.2974T>A	c.[1284+2T>C(+)2974T>C]	c.[3112C>T]+[3112C>T]
Family Hx		-	-	-	+	-
Age of onset		18	18	18	34	37
U/Ex	proximal	4+	4+	5	4	5
	distal	4+	3	5	4-	5
L/Ex	proximal	5	2	4	4+	5
	distal	*4*	2	4	2	4-
ECG		NSR	NSR, LVH, LAFB	NSR	NSR, LVH	NSR

U/Ex = upper extremity, L/Ex = lower extremity, ECG = electrocardiography, NSR = normal sinus rhythm, LVH = left ventricular hypertrophy, LAFB = left anterior fascicular block.

### ECG findings

All patients were in sinus rhythm. Left ventricular hypertrophy (LVH) was present in two patients (patients #2 and #4). In patient #2, left anterior fascicular block was also detected.

### Standard echocardiographic measurements

The data from standard echocardiography are summarized in Table 2. All subjects had preserved ejection fraction. Patient #4 showed mildly decreased left ventricular systolic function (LVEF = 45%) and hypokinesia of the basal and mid inferior wall. Echocardiographic findings of the other patients were unremarkable.

**Table 2:** Echocardiographic parameters in patients with Miyoshi myopathy

	#1	#2	#3	#4	#5
LVEDD (mm)	50	54	47	56	41
LVESD (mm)	30	31	30	44	25
Ejection Fraction (%)	64	67	59	45	63
E velocity (m/sec)	0.91	0.89	0.80	0.42	0.64
A velocity (m/sec)	0.54	0.51	0.60	0.61	0.66
E/A ratio (m/sec)	1.69	1.75	1.33	0.69	0.97
E' velocity (m/sec)	0.11	0.10	0.09	0.05	0.073
E/E' ratio	8.27	8.9	8.89	8.40	8.77
RWMA	(-)	(-)	(-)	Hypokinesia of basal and mid inferior wall	(-)
Global PSLS (%)	-15.7	-20.9	-17.4	-16.8	-23.4
PSLS at long-axis view (%)	-12.8	-19.3	-17.1	-17.0	-24.6
PSLS at 4-chamber view (%)	-16.8	-20.9	-18.9	-16.0	-22.7
PSLS at 2-chamber view (%)	-17.4	-22.7	-16.2	-17.3	-22.9
Segmental PSLS					
Basal Anterior	-22	-15	-24	-15	-22
AS	-15	-18	-16	-13	-18
IS	-17	-18	-16	-12	-18
Inferior	-17	-18	-23	-9	-25
IL	4	-17	-19	-14	-25
AL	-18	-11	-20	-12	-26
Mid Anterior	-14	-13	-24	-17	-22
AS	-18	-19	-17	-22	-25
IS	-19	-18	-18	-15	-21
Inferior	-19	-19	-21	-12	-24
IL	-11	-15	-21	-13	-25
AL	-15	-9	-21	-19	-22
Apical anterior	-15	-16	-21	-23	-25
septum	-20	-21	-23	-25	-28
Inferior	-20	-29	-22	-18	-25
lateral	-15	-17	-23	-25	-24
Apical cap	-17	-18	-22	-23	-26

LVEDD = left ventricular end-diastolic diameter, LVESD = left ventricular end-systolic diameter, E = peak transmitral flow velocity during early diastole, A = peak transmitral flow velocity during late diastole, E'= peak mitral annular velocity during early diastole, RWMA = regional wall motion abnormality, PSLS = peak systolic longitudinal strain, AS = anteriorseptum, IS = inferiorseptum, IL = inferiorlateral, AL = anteriorlateral.

### 2D strain by AFI

**Figure 1:**
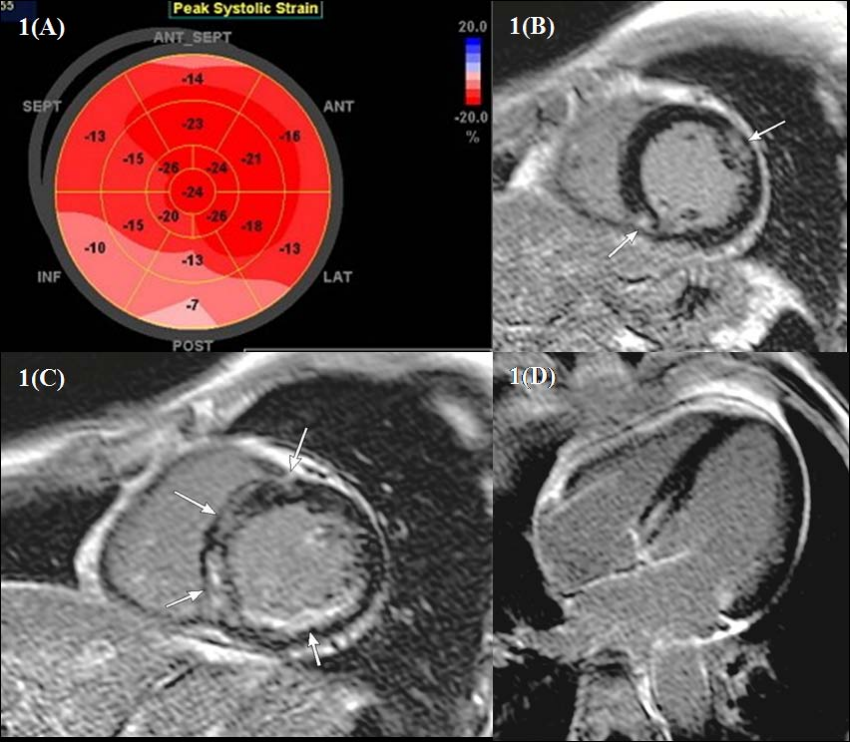
**Patient #4: (A) Automated function imaging (AFI) segmental PSLS by Echocardiography**. Segmental PSLS was decreased in inferior and basal anteroseptal area. (**B-D) Cardiovascular Magnetic Resonance. **(B,C) Late gadolinium enhancement images in the short-axis view (delay 10 minutes). There was fibrosis in inferior and basal anteroseptal area (arrow). (C) Late gadolinium enhancement images in the four-chamber view (delay 20 minutes). There was fibrosis in inferoseptal area (arrow).

Table 2 shows the PSLS measured by AFI. Although all five patients had preserved left ventricular systolic function, segmental PSLS was significantly decreased in patients #1, #2, and #4. In patient #1, the segmental PSLS was decreased in the basal to mid inferolateral wall. In patient #2, the segmental PSLS was decreased in the basal to mid anterolateral wall. In patient #3, the segmental PSLS was near normal. In patient #4, the segmental PSLS was decreased in inferior and basal anteroseptal wall. In patient #5, segmental PSLS was normal (Figure 1(A)).

### Comparison between patients with MM and age-matched healthy control subjects

There were no difference between patients with MM and age-matched healthy controls regarding age, sex, blood pressure, and heart rate. Echocardiographic parameters, including LVEDD, LVESD, LVEF, E, A, E/A ratio, E', and E/E' ratio were not significantly different between MM patients and control subjects. For 2D strain parameters, global PSLS was significantly lower in patients with MM than in control subjects (p = 0.005). Basal anterior septum PSLS, basal inferior septum PSLS, mid anterior PSLS, and mid inferior septum PSLS were significantly lower in patients with MM than in control subjects (P < 0.0001, < 0.0001, 0.038 and 0.003, respectively) (Table 3).

**Table 3:** Comparison between in patients with Miyoshi myopathy and age-matched health control subjects

	Patients with MM (n = 5)	Control (n = 5)	p-value
Age	43.8 ± 12.7	43.8 ± 12.7	1.0
Sex(Female)	2	2	1.0
SBP	126.8 ± 28	124.8 ± 18	0.883
DBP	81.6 ± 19	75.0 ± 10	0.527
HR	75.2 ± 14	69.8 ± 7.8	0.423
LVEDD (mm)	49.6 ± 5.9	49.2 ± 3.3	0.888
LVESD (mm)	32.0 ± 7.1	29.6 ± 3.2	0.518
Ejection Fraction (%)	59.6 ± 8.6	63.5 ± 3.8	0.379
E velocity (m/sec)	0.73 ± 0.20	0.85 ± 0.06	0.239
A velocity (m/sec)	0.58 ± 0.06	0.57 ± 0.05	0.777
E/A ratio (m/sec)	1.29 ± 0.46	1.51 ± 0.21	0.347
E' velocity (m/sec)	0.08 ± 0.02	0.09 ± 0.01	0.529
E/E' ratio	8.64 ± 0.29	9.61 ± 1.56	0.209
Global PSLS (%)	-18.8 ± 3.2	-24.4 ± 1.8	**0.005**
PSLS at long-axis view (%)	-18.2 ± 4.3	-24.1 ± 1.9	**0.017**
PSLS at 4-chamber view (%)	-19.1 ± 2.8	-22.9 ± 2.9	**0.019**
PSLS at 2-chamber view (%)	-19.3 ± 3.2	-24.4 ± 2.7	**0.010**
Segmental PSLS			
Basal Anterior	-19.6 ± 4.3	-23.4 ± 1.7	0.102
AS	-16.0 ± 2.1	-22.5 ± 1.1	<**0.0001**
IS	-16.2 ± 2.5	-23.4 ± 1.2	<**0.0001**
Inferior	-18.4 ± 6.2	-22.3 ± 1.6	0.213
IL	-14.2 ± 1.9	-21.9 ± 2.9	0.162
AL	-17.4 ± 6.1	-22.6 ± 2.6	0.106
Mid Anterior	-18.0 ± 4.8	-23.4 ± 2.3	**0.038**
AS	--20.2 ± 3.3	-22.1 ± 2.2	0.248
IS	-18.2 ± 2.2	-22.9 ± 2.2	**0.003**
Inferior	-19.0 ± 4.4	-22.4 ± 2.4	0.137
IL	-17.0 ± 5.8	-21.9 ± 1.7	0.109
AL	-18.6 ± 5.6	-23.2 ± 2.8	0.164
Apical anterior	-20.0 ± 4.4	-22.9 ± 2.7	0.206
septum	-23.4 ± 3.2	-23.1 ± 2.8	0.86
Inferior	-22.8 ± 4.3	-22.7 ± 2.6	0.947
lateral	-20.8 ± 4.5	-21.7 ± 2.2	0.684
Apical cap	-21.2 ± 3.7	-22.6 ± 2.5	0.463

SBP = systolic blood pressure, DBP = diastolic blood pressure, HR = heart rate, LVEDD = left ventricular end-diastolic diameter, LVESD = left ventricular end-systolic diameter, E = peak transmitral flow velocity during early diastole, A = peak transmitral flow velocity during late diastole, E'= peak mitral annular velocity during early diastole, PSLS = peak systolic longitudinal strain, AS = anterior septum, IS = inferior septum, IL = inferior lateral, AL = anterior lateral.

### LGE by CMR

The median inversion times for delayed myocardial imaging were 220 ms, 270 ms, and 310 ms for 5-minute, 10-minute, and 15-minute delayed scan, respectively. The detailed measurement data are shown in Table 4. The results of LGE by CMR were abnormal in four patients. Patient #1 showed mid anterior and anterolateral segment with myocardial fibrosis. Patient #2 had focal fibrosis in the basal to mid anterolateral wall. Patient #3 did not have evidence of myocardial fibrosis. Patient #4 had fibrosis in inferior area and mid wall fibrosis in basal anteroseptal wall (Figure 1(B-D)). Patient #5 had focal fibrosis in the septal side of the left ventricle. (See additional file 1 and 2: Movie for LGE in CMR).

**Table 4:** The results of CMR in patients with Miyoshi myopathy

	#1	#2	#3	#4	#5
**fibrosis**	**Mid anterior, mid anterolateral**	**Basal to mid inferolateral**	**No evidence of myocardial fibrosis**	**Basal to mid inferior, basal anteroseptal**	**septal side**
LVEF (%)	53.7	53.8	53.7	41.4	59.7
Stroke volume (ml)	61.9	73.9	44.2	43.5-	44.3
Cardiac output (l/min)	4.3	6.4	2.9	3.04-	2.5 l
LVEDV (ml)	115.3	137.3	82.2	156.03	74.16
LVESV (ml)	53.4	64.5	38.1	91.49	29.9
LVEDWM (g)	80.1	169.8	47.9	112.89	49.56

LVEF = left ventricular ejection fraction, LVEDV = left ventricular end-diastolic volume, LVESV = left ventricular end-systolic volume, LVEDWM = Left ventricular end-diastolic wall mass.

## Discussion

The main finding of our study is patients with MM showed subclinical involvement of the heart. Echo with 2D strain and LGE are sensitive methods of assessing the presence of myocardial dysfunction prior to the development of any cardiovascular symptoms. Miyoshi myopathy (MM) is an autosomal recessive distal myopathy characterized by early adult onset. MM is believed to spare the heart. However, recently, few case of cardiac involvement in MM reported. In our study, subclinical cardiac involvement was detected by 2D stain and LGE. Although most of patients had preserved LVEF, segmental PSLS was decreased in three patients by 2D strain and fibrosis was shown in four patients by LGE. In patient #1, there was discrepancy the abnormality between 2D strain and LGE. 2D strain was decreased in the basal to mid inferolateral area, but there were fibrosis in mid anterolateral area in LGE. In patient #2, segmental PSLS was decreased in the basal to mid anterolateral wall and there was focal fibrosis in same area. In patient #3, 2D strain and LGE were normal. In patient #4, 2D strain was decreased in inferior and basal anteroseptal area and there were fibrosis in inferior and mid wall basal anteroseptal area. Particularly in patient #4, it is reasonable that the abnormal results in the inferior area could be related to ischemic heart disease. Cardiac CT angiography showed non-significant stenosis (about 40%) in the right coronary artery. Considering the result of cardiac CT angiography, abnormality of the anteroseptal area was not be related to ischemic heart disease. The reduced PSLS detected by 2D strain tended to be in the same area as that which showed fibrosis in LGE in patient #2 and #4 among 5 patients. In patient #5, there was only focal septal fibrosis in LGE with normal 2D strain. It seems like nonspecific septal fibrosis in patient #5.

Myocardial strain imaging is useful for quantitative assessment of regional wall motion of the left ventricle. In our study, myocardial strain profile in MM patients with apparently normal 2-dimensional echocardiography were abnormal. Myocardial strain abnormalities are prevalent MM patients despite normal EF. Therefore, abnormal strain value would be early indicators of abnormal cardiac function in MM. Detection of such strain abnormalities might allow a better identification of MM cardiac dysfunction and might provide useful surrogate index to assess therapeutic efficacy. MM patients should be carefully evaluated for cardiac involvement using by conventional echocardiography, 2D strain and/or CMR. Any cardiac dysfunction should be promptly treated according to current guidelines. If the treatment were effective, decreased cardiac strain profile might be improved in follow up study. Further studies with long-term follow-up with a larger patient population are needed to determine the role of myocardial strain in preclinical diagnosis of myocardial impairment in MM. In other myopathies, such as Duchenne muscular dystrophy (DMD), patients with normal LV function showed myocardial strain profiles at the posterolateral wall of the LV that were different from those in healthy controls, suggesting abnormal myocardial contraction [7]. An earlier report demonstrated that in young patients with DMD who have globally normal systolic function, reductions in systolic deformation parameters could be detected in the anterolateral and inferolateral wall [8]. 2D strain appears to detect early changes in myocardial function in DMD patients before the onset of overt cardiomyopathy.

CMR has better sensitivity and could be used to assess ventricular size and function as well as to evaluate the characteristics of myocardial tissue. Specifically, LGE is used to evaluate myocardial fibrosis, which is present in dystrophinopathy patients. Silva *et al *[9] demonstrated LGE in seven of 10 patients with Duchenne and Becker muscular dystrophy. Recently, Puchalski *et al *[10] demonstrated that LGE was present in the basal inferolateral left ventricular free wall in a subepicardial distribution and extended to the basal inferior and basal anterolateral regions. These findings are consistent with necropsy studies that showed that the posterobasal abnormality spread to the epicardial third of the contiguous lateral LV free wall, with progressive transmural fibrous replacement and relative sparing of the right ventricle and ventricular septum [11,12].

The impact of subclinical cardiac involvement in MM remains to be assessed. Importantly, most MM patients may remain asymptomatic for years in spite of the progression of cardiac dysfunction because of their limited daily activities. Early detection of latent myocardial involvement before heart failure symptoms appear would be beneficial for delaying the progression of heart failure in MM. Therefore, follow-up is required. The findings of 2D strain and LGE are almost identical in MM patients. Thus, 2D strain and LGE could suggest subclinical early cardiac involvement and could be a noninvasive diagnostic screening test for evaluation of cardiac involvement in MM patients. Early recognition of cardiac involvement in MM is warranted, because adequate and appropriate therapy influences the further management and disease course of patients with primary myopathies [13].

There are several limitations to our study. First, our sample size was small. Although additional studies would be necessary to establish further the cardiac involvement in MM, the rarity of the disease may preclude a large-scale trial. Second, both of patient #2 and #4 had a history of hypertension and diabetes mellitus (DM). The durations of DM for patients #2 and #4 were 10 years and 22 years, respectively. It is possible that the cardiac involvement is not associated with the primary myopathy but rather with other risk factors, such as DM and hypertension. In patient #4, cardiac CT angiography showed mild stenosis (about 40%) in the right coronary artery. Considering the result of cardiac CT angiography, abnormality of the anteroseptal area was not related to ischemic heart disease. To exclude coronary artery disease, coronary angiography should be performed. Third, we did not obtain cardiac biopsies for definite confirmation of cardiac involvement in the MM patients.

## Conclusion

2D strain echo and LGE CMR were abnormal in Miyoshi myopathy patients with preserved left ventricular function and no overt heart failure symptoms. 2D strain and LGE could constitute a new diagnostic test to detect preclinical cardiac involvement in Miyoshi myopathy patients before the onset of overt cardiomyopathy.

## Competing interests

The authors declare that they have no competing interests.

## Authors' contributions

ERC carried out the studies and drafted the manuscript, SJP assisted in conception and design, data analysis and interpretation and drafting of the manuscript, YHC helped with conception and design, revising it critically for important intellectual content, DRR, SAC and JOC provided analysis and interpretation of data, SCL, SWP, BJK, DKK and JKO revised the manuscript critically for important intellectual content. All authors read and approved the final manuscript.

## Supplementary Material

Additional file 1 Cardiovascular Magnetic resonance - Late gadolinium enhancement inversion recovery images in the short-axis view. Click here for file

Additional file 2 Cardiovascular Magnetic resonance - Late gadolinium enhancement inversion recovery images in the short-axis view. Click here for file
